# Accident vasculaire cérébral hémorragique du tronc cérébral suite à une éclampsie du post partum: à propos d’un cas et revue de la littérature

**DOI:** 10.11604/pamj.2017.27.266.12288

**Published:** 2017-08-09

**Authors:** Khadija Ennaqui, Assia Makayssi, Houssine Boufettal, Naima Samouh

**Affiliations:** 1 Centre Hospitalier Universitaire Ibn Rochd, Faculty of Medecine and Pharmacy, Hassan II University of Casablanca, Morocco

**Keywords:** Eclampsie, accident vasculaire cérébral hémorragique, tronc cérébral, Eclampsia, stroke, brainstem

## Abstract

Les accidents vasculaires cérébraux sont des accidents rares mais graves durant la grossesse et le post-partum, ils sont responsable d'une mortalité et d'une morbidité élevée, la pré éclampsie, l'éclampsie et les accidents vasculaires cérébraux sont significativement liés. Le dysfonctionnement endothélial, l'altération de l'autorégulation cérébrale, et l'hypertension sévère sont, probablement, la cause de nombreux accidents vasculaires cérébraux chez la femme enceinte et durant la période du post-partum. Nous rapportons le cas d'un accident vasculaire cérébral hémorragique du tronc cérébral chez une patiente de 21 ans admise pour prééclampsie sévère sur grossesse non suivie de 38 SA compliquée d'une éclampsie puis d'un accident vasculaire cérébral hémorragique du tronc cérébral à j2 du post-opératoire d'une césarienne pour suspiçion d'hématome rétro-placentaire dès son admission. L'hémorragie intracérébrale est une complication rare mais grave chez les femmes en post-partum, plusieurs études sont en cours pour combler les lacunes de connaissances susceptibles d'être impliqués dans les soins des patientes prééclamptiques présentant des complications neurologiques.

## Abstract (en)

Strokes are rare but severe accidents during pregnancy and post-partum period. They are responsible for high mortality and morbidity rates. Pre-eclampsia, eclampsia, and strokes are significantly related. Endothelial dysfunction, alteration of brain self-regulation and severe hypertension are, probably, the cause of many strokes in pregnant women and during postpartum period. We report a case of hemorrhagic stroke of the brainstem in a nonmonitored 21-year old patient at 38 weeks pregnant admitted with severe preeclampsia complicated by eclampsia and then by haemorrhagic stroke of the brainstem on the second post-operative day after cesarean section performed due to suspicion of retroplacental haematoma arisen since her admission. Intracerebral hemorrhage is a rare but severe complication in post-partum women. Several studies are underway to fill the gaps of knowledge involved in the management of pre-eclamptic patients with neurological complications.

## Introduction

La pré éclampsie et une maladie systémique spécifique de la grossesse affectant 2 à 10% des grossesses [1], elle se définit comme l'apparition de novo d'une tension artérielle élevée (Tension artérielle systolique ≥ 140 mmHg et/ou tension artérielle diastolique ≥ 90 mmHg) associée à une protéinurie (≥ 300 mg/24H) ou sans protéinurie mais avec la présence d'œdème pulmonaire aigue et / ou d'insuffisance rénale aigue et/ou cytolyse hépatique et/ou thrombopénie, après 20 semaines de gestation [2]. L'éclampsie se définit par la survenue de convulsions chez une femme atteinte de pré-éclampsie. L'association entre l'éclampsie et l'hémorragie cérébrale a été reconnu depuis 1881 [3], Les accidents vasculaires cérébraux sont responsables de 50% des décès chez le pré éclamptique [4]. Nous rapportons l'observation d'une parturiente ayant eu une éclampsie du post-partum compliquée d'Accident vasculaire cérébral hémorragique du tronc cérébral. Le but de ce travail est de rapporter les particularités de cette complication exceptionnelle de l'éclampsie afin d'assurer une prise en charge rapide et adéquate.

## Patient et observation

**Figure 1:**
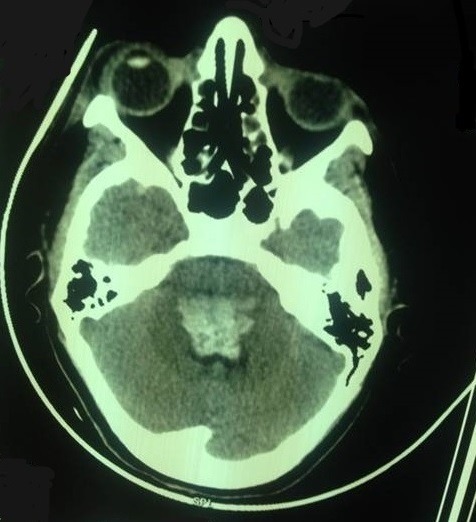
TDM cérébrale: coupe axiale après injection de produit de contraste montrant un hématome bulbo-protuberentiel mesurant 32/21/29 mm avec effet de masse sur les citernes de base

**Figure 2:**
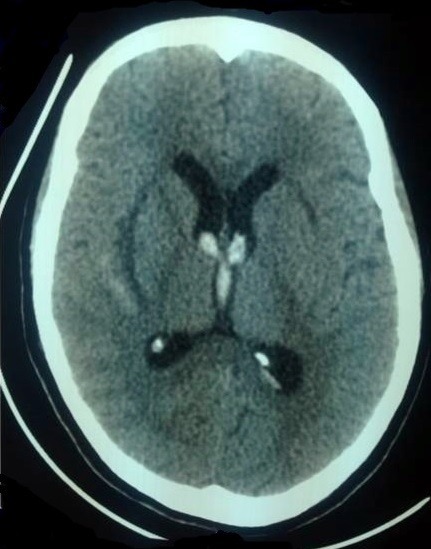
Hémorragie méningée en pariétal droit, hémorragie intra-ventriculaire au niveau des ventricules latéraux et du 4^ème^ ventricule

S.M, parturiente de 21 ans, sans antécédents pathologiques particuliers admise pour pré éclampsie sévère sur grossesse non suivie de 38 semaines d'aménorrhée, l'examen à l'admission retrouvait une patiente consciente 15/15 score de Glasgow, Tension artérielle: 150/100 mmHg, FC à 90 battements par minute, la fréquence respiratoire était à 18 cycle par minute, signes neurologiques de gravité: céphalées, bourdonnement d'oreilles, réflexes ostéo-tendineux vifs ainsi que des douleurs abdominales type barres épigastriques. L'examen obstétricale avait objectivé une hauteur utérine à 26 cm, avec un utérus tendu , bruits cardiaques fœtaux perçus à 100 battements par minute, au toucher vaginal, le col était en voie d'effacement dilaté à un cm, présentation céphalique ,poche des eaux intact, un bassin et un périnée sans particularités avec un doigtier souillé par un saignement noirâtre minime, devant ce constat un hématome rétro-placentaire fut suspecté pour lequel la patiente a été admise directement au bloc opératoire, elle avait bénéficié d'une mise en condition, d'un traitement antihypertenseur, d'une dose de charge de sulfate de Magnésium, d'un bilan biologique, en parallèle ,une césarienne était réalisée en urgence permettant l'extraction d'un nouveau né de sexe féminin, Apgar 8/10 à la 5^ème^ minute, d'un poids de naissance de 2250g avec la mise en évidence d'un HRP de 400 gramme, le bilan biologique avait objectivé un taux d'Hb à 13g/dl, un taux de plaquettes à 339000, TP à 71%, TCA à 35 seconde, Urée à 0.31g/l, Créatininémie à 9.8mg/l, ASAT à 462, ALAT à 286 UI/L et LDH à 1400g/l. Au cours de la surveillance, la patiente présentait une crise tonico-clonique généralisée suivie d'un coma postcritique, patiente transférée immédiatement au service de réanimation de la maternité ou elle fut intubée ventilée sédatée, l'examen à son admission en réanimation avait retrouvé une patiente inconsciente, stable sur le plan hémodynamique, la numération sanguine montrait une Hb à 7.6, des Plaquettes à 119000, une cytolyse hépatique ASAT à 700 UI/L, ALAT à 307 UI/L, une fonction rénale: Urée à 0.34 g/l, créatinine à 11.4 mg/l et LDH à 2080. Patiente était transfusée par 2 culots globulaire, mise sous protection gastrique, traitement antihypertenseur ainsi que la dose d'entretien du sulfate de Mg2+ en SAP et une protection contre la maladie thromboembolique par des bas de contention, à H24 d'hospitalisation en réanimation et devant le retard de réveil, un scanner cérébral était réalisé objectivant un hématome bulbo-protubérantiel mesurant 32*21*29mm avec effet de masse sur les citernes de base (Figure 1), hémorragie méningée pariétale droite et hémorragie intra ventriculaire ventricules latéraux et du 4^ème^ ventricule (Figure 2). L'évolution était marquée par l'installation d'une instabilité hémodynamique à H30 du postopératoire, mise sous noradrénaline 2mg/heure, puis la survenue d'un arrêt cardiaque non récupéré malgré les mesures de réanimation puis la patiente était déclarée décédée à H 34 du postopératoire.

## Discussion

L'accident vasculaire cérébral est une urgence neurologique à morbi-mortalité très élevée, des études récentes ont montré que l'incidence de l'accident vasculaire cérébral, bien que rare, est en augmentation chez les femmes enceintes et en post-partum. En 2011, Kuklina et al [4, 5] une recherche publiée qui analysait les données du registre national d'hospitalisation, entre 1994-1995 et 2006-2007, les taux d'hospitalisations pour accident vasculaire cérébral ont augmenté respectivement de 47% et 83%. En résumant plusieurs études, le taux d'accident vasculaire cérébral est estimé à 25-34 Cas pour 100 000 accouchements, alors que l'incidence de l'accident vasculaire cérébral chez les femmes non enceintes 15-44 ans est de 11 pour 100.000 femmes [6, 7]. L'étude de Kuklina et al rapporte également que les troubles hypertensifs ont été directement impliqués dans les accidents vasculaires cérébraux pendant la grossesse. D'autres études ont montré des résultats similaires [7, 8]. L'hypertension est reconnue comme étant le premier facteur de risque d'accident vasculaire cérébral, et ce n'est pas une exception chez la femme enceinte. Hypertension artérielle durant la grossesse peut être préexistante, gestationnelle ou secondaire à la pré-éclampsie ou à l'éclampsie, comparativement aux femmes sans hypertension, les femmes souffrant d'HTA au cours de la grossesse sont six à neuf fois plus susceptible d'avoir un accident vasculaire cérébral [8]. D'autres facteurs de risques ont été décrits durant la grossesse à savoir : le diabète, les valvulopathies, les maladies de système, l'hypercoagulabilité, le changement de l'architecture artérielle cérébrale entraînant une diminution du collagène, de l'élasticité des vaisseaux, et de ces faits l'identification des facteurs de risque d'accident vasculaire cérébral pendant la grossesse est essentielle pour prévenir cette situation rare et souvent grave. L'accident vasculaire cérébral hémorragique touche également les femmes enceintes. Il peut se manifester par des maux de tête intenses, une raideur du cou, des nausées et vomissements, des troubles de conscience, des convulsions ou des anomalies neurologiques focales.

L'évaluation initiale inclue un scanner cérébral sans injection de produit de contraste, c'est l'examen radiologique de choix pour mettre en évidence une hémorragie intracérébrale sous forme de pétéchies diffuses au niveau du cortex, de petites aires hémorragiques sous corticales ou d'hématomes, l'angioIRM peut être réaliser afin d'identifier la source de l'hémorragie, la mise en évidence d'un anévrysme ou d'une malformation artério-veineuse nécessitera une prise en charge par les neurochirurgiens. L'hypertension intracrânienne dans le cadre de l'éclampsie doit être étroitement gérée par les antihypertenseurs et les anticonvulsivants, y compris le sulfate de magnésium [9, 10]. Les lésions hémorragiques présentent un pronostic particulièrement défavorable [11]. Elles sont responsables de 40 à 60% des décès éclamptiques. Sur une série de 18 patientes de STAUDER, deux patientes ayant un syndrome hémorragique ont évolué favorablement et une patiente avait présenté des séquelles visuelles à distance de l'épisode éclamptique [12]. L'œdème cérébral diffus parait être également un facteur de mauvais pronostic. Hibbard, 1973 et Lopez Liera et al, 1976, ont constaté la présence d'un œdème cérébral chez 20% de femmes décédées d'éclampsie [11, 12]. La crise éclamptique est le plus souvent une complication des grossesses mal suivies. La stratégie la plus efficace pour détecter la pré-éclampsie est de surveiller les chiffres tensionnels pendant le deuxième et le troisième trimestre de la grossesse. Plusieurs agents thérapeutiques sont utilisés comme des mesures préventives de l'éclampsie. Actuellement, le traitement par l'aspirine a montre son efficacité en termes de prévention. En effet, une étude prospective randomisée avait montre une diminution significative du taux de pré éclampsie dans le groupe de femmes ayant des antécédents obstétricaux majeurs traitées par 150 mg/j d'aspirine et 300 mg/j .En pratique, on utilise des doses de 50 a 100 mg /j a la 14^ème^ semaine s'il existe des antécédents pathologiques ou a la 22^ème^ semaines si le doppler est pathologique [13].

## Conclusion

L'éclampsie reste encore fréquente dans les pays en voie de développement. C'est une complication grave du pré éclampsie, responsable d'une mortalité maternelle et infantile élevée. La physiopathologie des lésions intracrâniennes est complexe associant encéphalopathie hypertensive et ischémie secondaire à une atteinte vasculaire endothéliale et à un vasospasme cérébral. La réalisation systématique de l'IRM avec des séquences de diffusion chez les éclamptiques ayant des troubles neurologiques permet une meilleure approche diagnostique et pronostique de ces patientes. Une prise en charge thérapeutique rapide est nécessaire afin de contrôler ces différents processus, éviter leur aggravation et obtenir un meilleur pronostic.

## Conflits d’intérêts

Les auteurs ne déclarent aucun conflit d'intérêt.
